# Glutamatergic Retinal Waves

**DOI:** 10.3389/fncir.2016.00038

**Published:** 2016-05-10

**Authors:** Daniel Kerschensteiner

**Affiliations:** Departments of Ophthalmology and Visual Sciences, Neuroscience, and Biomedical Engineering, Hope Center for Neurological Diseases, Washington University School of MedicineSaint Louis, MO, USA

**Keywords:** retina, development, spontaneous activity, visual system, synaptic refinement, asynchronicity

## Abstract

Spontaneous activity patterns propagate through many parts of the developing nervous system and shape the wiring of emerging circuits. Prior to vision, waves of activity originating in the retina propagate through the lateral geniculate nucleus (LGN) of the thalamus to primary visual cortex (V1). Retinal waves have been shown to instruct the wiring of ganglion cell axons in LGN and of thalamocortical axons in V1 via correlation-based plasticity rules. Across species, retinal waves mature in three stereotypic stages (I–III), in which distinct circuit mechanisms give rise to unique activity patterns that serve specific functions in visual system refinement. Here, I review insights into the patterns, mechanisms, and functions of stage III retinal waves, which rely on glutamatergic signaling. As glutamatergic waves spread across the retina, neighboring ganglion cells with opposite light responses (ON vs. OFF) are activated sequentially. Recent studies identified lateral excitatory networks in the inner retina that generate and propagate glutamatergic waves, and vertical inhibitory networks that desynchronize the activity of ON and OFF cells in the wavefront. Stage III wave activity patterns may help segregate axons of ON and OFF ganglion cells in the LGN, and could contribute to the emergence of orientation selectivity in V1.

Retinal waves have been observed in many species including primates (Warland et al., 2006). In evolution, waves seem to have emerged as a source of patterned activity in species that refine ganglion cell projections while visually deprived inside a shell or womb (Demas et al., 2012). Consistent with this idea, waves have been found in all amniotes tested, but not in amphibians, which use vision to find food and avoid predators as soon as ganglion cell axons reach their targets (Holt and Harris, 1983; Demas et al., 2012). In this review, I will focus on recent data obtained in mice. An excellent review of earlier work in ferrets, chickens, and turtles can be found here (Wong, 1999).

## Patterns of Glutamatergic Retinal Waves

Glutamatergic waves pervade the mouse retina from postnatal day 10–14 (stage III, P10–P14). They are preceded by cholinergic waves (stage II, P1–P10) and localized bursts of activity mediated by gap-junctional coupling among nearby ganglion cells (stage I, embryonic day 17–P1; Bansal et al., 2000; Demas et al., 2003; Blankenship and Feller, 2010; Maccione et al., 2014). The beginning of stage III waves and the end of stage II waves appear to be mechanistically linked; and cholinergic waves persist when glutamatergic waves are disrupted (Blankenship et al., 2009; Xu et al., 2016); and precocious glutamatergic waves are observed when cholinergic waves are disrupted (Bansal et al., 2000; Xu et al., 2016). Glutamatergic waves subside around the time of eye opening (P14) as light-evoked signals begin to drive retinal activity. The disassembly of glutamatergic waves requires normal signaling between photoreceptors and bipolar cells, but occurs independent of visual experience (Demas et al., 2003, 2006).

In waves of all stages, bursts of ganglion cell activity spread across the retina (Meister et al., 1991). Based on large-scale multielectrode array recordings and calcium imaging *in vitro*, glutamatergic waves are estimated to spread laterally at 150–200 μm/s (Blankenship et al., 2009; Maccione et al., 2014). Compared to stage II waves (0.5–1 mm^2^), individual stage III waves encompass smaller areas of the retina (~0.2 mm^2^; Maccione et al., 2014). In each glutamatergic wave, ganglion cells fire 2–5 bursts of action potentials lasting ~0.6 s per burst; and consecutive waves are separated by ~60 s of silence (Demas et al., 2003; Kerschensteiner and Wong, 2008; Blankenship et al., 2009; Maccione et al., 2014). A unique feature of glutamatergic waves is the asynchronous recruitment of ganglion cells that respond to light increments (ON) and decrements (OFF), respectively. In each wavefront, neighboring ON and OFF ganglion cells fire in sequence: ON before OFF (Kerschensteiner and Wong, 2008; Akrouh and Kerschensteiner, 2013). Cross-correlations of ON and OFF ganglion cell spike trains peak at 0.8–1 s, indicating that opposite sign cells fire temporally adjacent non-overlapping bursts, whereas same sign pairs (i.e., ON—ON and OFF—OFF) fire synchronously with cross-correlations peaking at 0 s (Kerschensteiner and Wong, 2008; Akrouh and Kerschensteiner, 2013).

In addition to spreading within the retina, waves propagate forward through the visual system. *In vivo* imaging in awake mice revealed that stage II waves dominate activity in superior colliculus (SC) and lateral geniculate nucleus (LGN), two major targets of ganglion cell axons (Ackman et al., 2012), and in primary visual cortex (V1), the primary target of LGN axons (Hanganu et al., 2006; Ackman et al., 2012). In addition to a preliminary report on forward propagation of stage III waves *in vivo* (Gribizis et al., 2015), one study found that a subset of spontaneous activity patterns in developing V1 (P10–P14) were suppressed by enucleation (Siegel et al., 2012). This suggests that glutamatergic retinal waves are relayed through the early visual system up to V1.

## Mechanisms of Glutamatergic Retinal Waves

Early in the exploration of waves, stage III ganglion cell activity was shown to rely on glutamatergic input (Wong et al., 2000); subsequently, bipolar cells were identified as the source of this input (Blankenship et al., 2009). However, the circuits that activate bipolar cells (i.e., wave initiation), spread activity laterally (i.e., wave propagation), and desynchronize the firing of neighboring ON and OFF ganglion cells (i.e., wave patterning), until recently, remained obscure.

**Figure 1:**
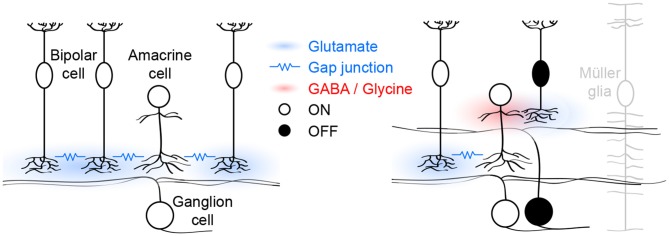
**Schematic of glutamatergic wave circuits.** The *left panel* illustrates the lateral excitatory (*blue*) network of ON cone bipolar and amacrine cells, which generates and propagates stage III waves. Activity spreads by lateral glutamatergic transmission and gap junctions among neighboring cells. The *right panel* illustrates the vertical inhibitory (*red*) pathway through which ON cone bipolar cells hyperpolarize OFF cone bipolar cells and delay excitatory input to and spiking off OFF ganglion cells. Müller glia limit the spread of glutamate and maintain temporal separation of ON and OFF activity.

The mouse retina has 13 bipolar cell types, which can be grouped into functional classes based on whether their dendrites preferentially contact rods (1 type) or cones (12 types). Cone bipolar cells can further be divided into those that respond to light increments (ON, 7 types) and those that respond to light decrements (OFF, 5 types; Euler et al., 2014). All bipolar cells release glutamate, which they package into vesicles via VGluT1 (Johnson et al., 2003); and stage III waves are abolished in VGluT1 knockout (*VG1 KO*) mice (Blankenship et al., 2009). A recent study found that ON cone bipolar, OFF cone bipolar, and rod bipolar cells contribute differently to glutamatergic waves (Akrouh and Kerschensteiner, 2013). I will use the responses of these functional bipolar cell classes to organize the following discussion of the circuit mechanisms that generate, propagate, and pattern stage III waves (Figure 1).

In each glutamatergic wave, ON cone bipolar cells depolarize, causing a transient increase in intracellular calcium and eliciting glutamate release onto ON ganglion cells, which as a result fire bursts of action potentials (Akrouh and Kerschensteiner, 2013; Firl et al., 2013). Electrophysiological “sniffer patch” recordings, and imaging studies using soluble and cell-surface-bound fluorescent reporters have shown that glutamate escapes from synaptic clefts and spreads extracellularly during stage III waves (Blankenship et al., 2009; Firl et al., 2013; Rosa et al., 2015). This excites a subset of developing ON cone bipolar cells that express ionotropic glutamate receptors (iGluRs) on their axons (Akrouh and Kerschensteiner, 2013). The remaining ON cone bipolar cells lack such receptors and are instead excited via gap junctions with nearby iGluR-expressing ON cone bipolar cells and/or amacrine cells (Akrouh and Kerschensteiner, 2013). Together, these two excitatory mechanisms, coordinated by extracellular glutamate, support the lateral propagation of glutamatergic waves (Figure 1, *left panel*). Pharmacological blockade of either iGluRs or gap junctions is sufficient to block stage III activity in ganglion cells and bipolar cells (Akrouh and Kerschensteiner, 2013). Interestingly, glutamatergic waves persist when the connexin subunits that make up bipolar cell gap junctions are deleted in the germline (Blankenship et al., 2011), a manipulation that may trigger compensatory changes in iGluR expression. Spontaneous network activity can either be initiated by pacemaker activity of individual neurons or by amplification of coincident membrane potential fluctuations in recurrent networks (Kerschensteiner, 2013). The observation that ON cone bipolar cell depolarizations are abolished when networks are pharmacologically uncoupled, argues against them functioning as pacemakers (Akrouh and Kerschensteiner, 2013). Similarly, AII amacrine cells, which are gap-junctionally coupled to ON cone bipolar cells (Lin et al., 2005; Marc et al., 2014), and which at maturity can generate rhythmic bursting (Cembrowski et al., 2012), do not function as pacemakers during glutamatergic waves (Firl et al., 2015). Together these findings suggest that the lateral excitatory networks that propagate glutamatergic retinal waves also initiate them by amplifying coincident fluctuations in the membrane potential of nearby ON cone bipolar and/or amacrine cells.

Imaging and electrophysiological studies both indicate that rod bipolar cells, which do not provide direct input to ganglion cells, are not reliably recruited into stage III waves (Akrouh and Kerschensteiner, 2013; Firl et al., 2013). This is likely because they are not integrated into the gap-junctional network of ON cone bipolar cells (Akrouh and Kerschensteiner, 2013). In addition, rather than iGluRs, rod bipolar cells express the excitatory amino acid transporter 5 (EAAT5) on their axon terminals, a glutamate uptake transporter with a large chloride conductance (Veruki et al., 2006; Wersinger et al., 2006; Ichinose and Lukasiewicz, 2012; Akrouh and Kerschensteiner, 2013).

As ON cone bipolar cells depolarize during stage III waves, OFF cone bipolar cells hyperpolarize (Akrouh and Kerschensteiner, 2013). This hyperpolarization results from dominant inhibitory synaptic inputs to the axon terminals of OFF cone bipolar cells by a combination of GABAergic and glycinergic amacrine cells with neurites that stratify diffusely in the inner plexiform layer (Akrouh and Kerschensteiner, 2013). Diffuse amacrine cells are depolarized by glutamatergic input from ON cone bipolar cells and form a vertical inhibitory pathway through which ON cone bipolar cells hyperpolarize OFF cone bipolar cells (Figure 1, *right panel*). Simultaneous recordings of bipolar cell voltage and excitatory input to ganglion cells, indicate that OFF cone bipolar cells release glutamate as their voltage returns to baseline from the wave-associated hyperpolarization (Akrouh and Kerschensteiner, 2013). Bipolar cell axons release glutamate at ribbon synapses, which enable them to adjust release continuously to gradual changes in voltage (Matthews and Fuchs, 2010). Why does the return to sustained baseline voltage elicit transient glutamate release from OFF cone bipolar cells? One possible explanation is that the readily releasable pool of vesicles in OFF cone bipolar cells is depleted between waves and replenished during the wave-associated hyperpolarization, causing a subsequent transient increase in release. This remains to be experimentally tested, but similar rapid changes in vesicle pool occupancy have been observed at mature bipolar cell synapses (Mennerick and Matthews, 1996; Burrone and Lagnado, 2000; Singer and Diamond, 2003, 2006) where they contribute to adaptive computations (Manookin and Demb, 2006; Dunn and Rieke, 2008; Jarsky et al., 2011; Oesch and Diamond, 2011). As waves propagate through lateral excitatory networks, ON cone bipolar cells thus engage vertical inhibitory pathways that hyperpolarize OFF cone bipolar cells. The synchronous opposite sign responses of ON and OFF cone bipolar cells are translated into a time-locked sequence of glutamate release and spiking of ON and OFF ganglion cells in the wavefront.

In the inner plexiform layer of the retina, ON and OFF circuits are separated vertically. For glutamate release from ON and OFF cone bipolar cells to stimulate ON and OFF ganglion cells sequentially, the vertical spread of glutamate needs to be restricted (Figure 1, *right panel*). DL-threo-beta-benzyloxyaspartate (TBOA), an antagonist of EAATs, synchronizes excitatory input to ON and OFF ganglion cells, indicating that EAAT-mediated uptake limits the spread of glutamate and is critical for patterning stage III waves (Akrouh and Kerschensteiner, 2013). Müller glia express EAAT1 and at maturity are the primary agent of glutamate uptake in the retina (Pow and Crook, 1996; Pow et al., 2000). Electrophysiological recordings from Müller glia showed that they depolarize during stage III waves (Akrouh and Kerschensteiner, 2013) possibly due to electrogenic glutamate uptake via EAAT1 (Owe et al., 2006). Moreover, a recent study identified calcium transients in Müller glia processes in the inner plexiform layer during stage III waves (Rosa et al., 2015). The function of these signals, which are mediated by iGluRs on Müller glia (Rosa et al., 2015), remains to be determined.

## Functions of Glutamatergic Retinal Waves

In this section, I will discuss how glutamate release from bipolar cells during stage III waves regulates circuit development in the retina, and how the patterns of ganglion cell activity, which propagate forward through the visual system, may shape wiring in retinorecipient structures (e.g., LGN and SC) and in V1.

As outlined in the previous section, ON and OFF cone bipolar cells release glutamate with each stage III wave. The effects of glutamate release from bipolar cells on circuit development in the retina have been analyzed in several recent studies. Glutamate release from ON cone bipolar cells was suppressed by transgenic expression of tetanus toxin (i.e., *TeNT* mice). In *TeNT* mice, ON cone bipolar and ON ganglion cells are connected by fewer synapses at maturity (Kerschensteiner et al., 2009). Live imaging showed that reduced connectivity is a result of lower rates of synapse formation, whereas synapse elimination is unaffected in *TeNT* mice (Kerschensteiner et al., 2009). Effects of release suppression are pathway specific; and excitatory synapses form normally on OFF ganglion cells and OFF dendrites of ON-OFF ganglion cells (Kerschensteiner et al., 2009). Furthermore, in ON ganglion cells that receive convergent input from multiple ON cone bipolar cell types, connectivity is reduced in a cell-type-specific manner in *TeNT* mice (Morgan et al., 2011). In a mouse model in which glutamate release is enhanced, rates of synapse formation are elevated with similar cell type specificity (Soto et al., 2012). When expression of tetanus toxin is limited to a sparse subset of ON cone bipolar cells, synapse formation on ON ganglion cell dendrites is reduced locally without competition among neighboring axons (Johnson and Kerschensteiner, 2014; Okawa et al., 2014). In addition to effects of glutamate release from bipolar cells on synapse formation between their axons and ganglion cells dendrites, retrograde plasticity was observed, in which axonal glutamate release affects the ability of bipolar cell dendrites to recruit input from photoreceptors (Johnson and Kerschensteiner, 2014). Whereas no differences in ganglion cell dendrite structure were observed in *TeNT* mice (Kerschensteiner et al., 2009), in CD3ζ knockout mice, which generate fewer stage III waves, the number of filopodia on ganglion cell dendrites was increased (Xu et al., 2010). This effect is phenocopied by intraocular injections of glutamate receptor blockers (Xu et al., 2010).

In LGN and SC, eye-specific segregation and retinotopic refinement of ganglion cell axons emerge during stage II waves (Kerschensteiner, 2013; Ackman and Crair, 2014). Waves promote this organization via burst-time-dependent plasticity as they synchronize activity of nearby ganglion cells in the same eye more than of ganglion cells further apart in the same eye or in different eyes (Grubb et al., 2003; McLaughlin et al., 2003; Butts et al., 2007; Shah and Crair, 2008; Xu et al., 2011; Ackman et al., 2012; Zhang et al., 2012; Burbridge et al., 2014). Stage III waves are critical for the maintenance of these wiring patterns. In no b-wave mice, in which stage III waves are replaced by high-frequency bursting, ganglion cell axons from the two eyes desegregate (Demas et al., 2006). Similarly, synchronous optogenetic stimulation of ganglion cells in both eyes during the period of stage III waves, disrupts eye-specific segregation and retinotopy (Zhang et al., 2012). Finally, in mice with disrupted stage II waves, stage III waves are able to support eye-specific segregation and retinotopic refinement, but this organization is lost when both stage II and III waves are abolished (Xu et al., 2016).

In addition to maintaining eye-specific and retinotopic organization, the patterns of glutamatergic waves may promote ON/OFF segregation in LGN (Kerschensteiner and Wong, 2008; Kerschensteiner, 2013). Given burst-time-dependent plasticity rules (Butts et al., 2007), the asynchronous recruitment of neighboring ON and OFF ganglion cells during stage III waves is expected to separate converging ON and OFF axons in retinotopically refined projections to LGN (Kerschensteiner and Wong, 2008). Although this remains to be directly tested, the following circumstantial evidence supports a role for glutamatergic waves in ON/OFF segregation in mouse LGN. First, in ferrets, pharmacologic blockade of stage III waves prevents ON/OFF segregation (Hahm et al., 1991; Cramer and Sur, 1997); and pharmacologic enhancement of stage III waves accelerates receptive field maturation (Davis et al., 2015). Second, artificial neuronal networks with plasticity rules observed in subcortical visual circuits (Butts et al., 2007; Shah and Crair, 2008) undergo reliable ON/OFF segregation in response to recorded retinal activity patterns (Gjorgjieva et al., 2009). Third, mice with precocious stage III waves exhibit excessive ON/OFF segregation: SC neurons, which normally respond to both ON and OFF stimuli, become purely ON or OFF responsive (Chandrasekaran et al., 2005); ON and OFF neurons in LGN, which normally intermingle, form clusters (Grubb et al., 2003). An interesting question in this context is why the same sequence of ganglion cell activity promotes ON/OFF segregation in LGN, but not in SC. The answer may lie in the different timing of critical periods of refinement in these circuits. In SC, synaptic remodeling appears complete by P7 (Chandrasekaran et al., 2005), before the onset of stage III waves, whereas in LGN, most early synaptic remodeling occurs between P11 and P14 (Hooks and Chen, 2006).

If stage III waves are faithfully transmitted to V1, they could contribute to the formation of orientation selective receptive fields. Orientation selectivity in V1 arises in part from the convergence of axons of ON and OFF LGN neurons with spatially offset receptive fields (Hubel and Wiesel, 1962; Lien and Scanziani, 2013; Niell, 2013). Spontaneous activity patterns that synchronize the activity of ON and OFF neurons at appropriate distances could help set up this organization (Miller, 1994). The centers of the ON and OFF portions of orientation-selective V1 receptive fields are ~5° apart (Lien and Scanziani, 2013), equivalent to ~170 μm in retinal space (Remtulla and Hallett, 1985; Schmucker and Schaeffel, 2004). Given propagation speeds of 150–200 μm/s (Blankenship et al., 2009; Maccione et al., 2014) and a temporal delay of 0.8–1 s between the firing of neighboring of ON and OFF ganglion cells (Kerschensteiner and Wong, 2008; Akrouh and Kerschensteiner, 2013), it is possible the stage III wave synchronize the activity of opposite sign LGN neurons at appropriate distances to drive their convergence in V1. If such spatially offset synchronization exists in glutamatergic waves and whether it contributes to orientation selectivity in V1 remains to be experimentally tested.

## Summary

Recent studies have described in detail the patterns of glutamatergic waves in the retina and have identified the circuit mechanisms that generate, propagate and shape this activity. Activity patterns observed in the retina appear well-suited to promote ON/OFF segregation in LGN and orientation selectivity in V1. However, forward propagation of stage III waves through the visual system remains to be clearly demonstrated; and *in vivo* manipulations that alter patterns of glutamatergic waves without affecting activity levels are needed to test their role in circuit organization in LGN and V1.

## Author Contributions

DK wrote the manuscript.

## Funding

Work of DK is supported by funding from the National Institutes of Health (NIH; EY021855, EY023341) and by a Career Development Award from the Research to Prevent Blindness Foundation.

## Conflict of Interest Statement

The author declares that the research was conducted in the absence of any commercial or financial relationships that could be construed as a potential conflict of interest.
